# Repellency Assessment of *Nepeta cataria* Essential Oils and Isolated Nepetalactones on *Aedes aegypti*

**DOI:** 10.1038/s41598-018-36814-1

**Published:** 2019-02-06

**Authors:** William Reichert, Jadrian Ejercito, Tom Guda, Xujun Dong, Qingli Wu, Anandasankar Ray, James E. Simon

**Affiliations:** 1 New Use Agriculture and Natural Plant Products Program, Department of Plant Biology, Rutgers University, New Brunswick, New Jersey USA; 2 Department of Molecular, Cell and Systems Biology, University of California Riverside, Riverside, California USA; 3 College of Life Science, Shaanxi Normal University, Xi’ an, Shaanxi China; 4 Department of Medicinal Chemistry, Ernest Mario School of Pharmacy, Rutgers University, Piscataway, New Jersey USA

## Abstract

There is an increased need for improved and affordable insect repellents to reduce transmission of rapidly spreading diseases with high mortality rates. Natural products are often used when DEET cannot be afforded or accessed and when consumers choose not to use a synthetic repellent. The essential oils from two newly bred *Nepeta cataria* (catnip) plants representing two different chemotypes and their respective isolated nepetalactone isomers were evaluated as mosquito repellents against *Aedes aegypti* mosquitoes that transmit the Zika and Dengue virus in a one choice landing rate inhibition assay. A dose response curve was generated for each treatment and a time course analysis of repellency was performed over 24 hours with a *N. cataria* essential oil sample. The results indicate that all essential oil samples and their respective purified nepetalactone isomers were able to achieve greater than 95% repellency. Between two and four hours, the ability to repel more than 95% of the mosquitoes diminished. At the lowest concentrations tested, the nepetalactones and crude essential oil samples were more effective than DEET at reducing the number of mosquito landings.

## Introduction

Mosquitoes vector the deadliest diseases on the planet killing an estimated half a million people annually by Malaria, Yellow fever and the Dengue virus alone^1–3^. Recently, a newer threat known as the Zika virus reemerged and is being rapidly spread by *Aedes aegypti* mosquitoes, as well as from a viremic mother to her newborn and by sexual intercourse throughout the western hemisphere, potentially causing neurological disorders and microcephaly^4^. Mosquitoes seek out hosts in search of a blood meal for reproduction providing an opportunity for the infectious agent to enter the host while feeding^5^. The common symptoms of all these diseases are rashes, a high fever and chills that all complicate emergency room diagnosis due to the similarities, resulting in a missed or delayed diagnosis that could result in mortality^6^. Some diseases such as the West Nile virus show very little symptoms and can go undiagnosed until movement loss or neurological illness^7^. Disease vectoring mosquitoes cover the world and efforts to control the spread of diseases, identify new repellents and to educate people lowering the risk of infection are currently being implemented on a multinational level^8–10^. DDT (1,1′-(2,2,2-Trichloroethane-1,1-diyl)bis(4-chlorobenzene)) is a pesticide that was used globally in the past to kill mosquitoes but its usage has been severely curtailed due to its negative environmental impacts^11^. Educating individuals about mosquito control in regions of the globe where there is a high rate of disease incidence on how to manage mosquito populations has been effective at reducing infections^12,13^.

Insect repellents developed by government and private industries are very effective at deterring mosquitoes and protecting the users from contracting these diseases^14^. DEET (N,N-Diethyl-3-methylbenzamide) has been the benchmark of insect repellents since its development for the United States Army to use in tropical regions where there is a high incidence of insect transmitted diseases^15^. While DEET is extremely effective, it is not as volatile as other insect repellents and leads to a limited spatial range of repellency for mosquitoes and other insects^16^. Whereas another critical concern associated with the consistent use of DEET is its potential toxicity. Studies have shown DEET inhibits human acetyl cholinesterase, modulates G-protein coupled receptors and inhibits ion channels^17–21^. Numerous publications exist urging caution in its use and claim that DEET is unsuitable for young children and pregnant females, though the Centers for Disease Control and Protection (CDC; Atlanta, GA) still recommends the use of it for vector protection^22–24^. DEET is also absorbed through the skin at a high rate and special formulations are required to reduce transdermal absorption^25^. However, other reviews suggest that it does not cause adverse health effects^26^. The costs of DEET have made it unaffordable to many of those living in regions affected by disease vectoring mosquitoes such as sub-Saharan Africa, China and India. Between the costs, access, and public concern about the dangers of DEET individuals and families could prefer locally sourced natural insect repellants derived from eucalyptus, mints, cloves, basils and neem leaves rather than DEET^27^.

While many regions of the world have access to DEET and other effective insect repellents, natural products still serve as a primary source of repellents in China, India and sub-Saharan Africa where people cannot afford or do not have access to them^27^. Specialty crops have been cultivated to produce a wide array of insect repellents in these same regions to repel disease-causing insects from natural sources dating back to before the Common Era^27,28^. Ethnobotanical resources have led to the identification of plants that can be used to repel insects^27,29^. Volatizing citronella and geraniol from lemongrass oil as well as neem oil are the most common natural sources of mosquito repellents^30,31^. In 2005, the CDC endorsed para-Menthane-3, 8-diol (PMD), a steam distillate product from the leaves of the Australian lemon-scented gum tree as a mosquito repellent^32^. Insect repellents derived from natural sources such as volatile essential oils have been shown to be efficient at repelling mosquitoes for up to an hour, however these chemicals are not as effective as DEET overall due to their limited duration of acceptable repellency^33–35^.

Natural insect repellent formulations using essential oils from aromatic plants in the Lamiaceae family are sourced from the glandular trichromes on the epidermis of leaves and flowers. Plants biosynthesize multiple compounds in their essential oil, not just a single desirable chemical and breeding programs have increased the bioactive compounds concentration within the essential oil of multiple species of plants across the kingdom^36–40^. Botanicals from the Lamiaceae family have also demonstrated that their essential oils can act as a mosquito repellent comparable to DEET^41,42^. One member of this family, *Nepeta cataria* (catnip), has had its essential oils containing various nepetalactone stereoisomers tested and the results showed that it is comparable to DEET at repelling mosquitoes, while offering better spatial repellency^43–45^. Nepetalactones are the distinguishing natural compounds associated with catnip and are the euphoria-inducing agent in felines responsible for their characteristic behavior^46^. Like many genera of the Lamiaceae plant family, the *Nepeta* genus contains species that produce a wide array of volatile compounds in their essential oil with nepetalactones, β-caryophyllene, nerol, citronellol and geraniol present^47–49^.

**Figure 1:**
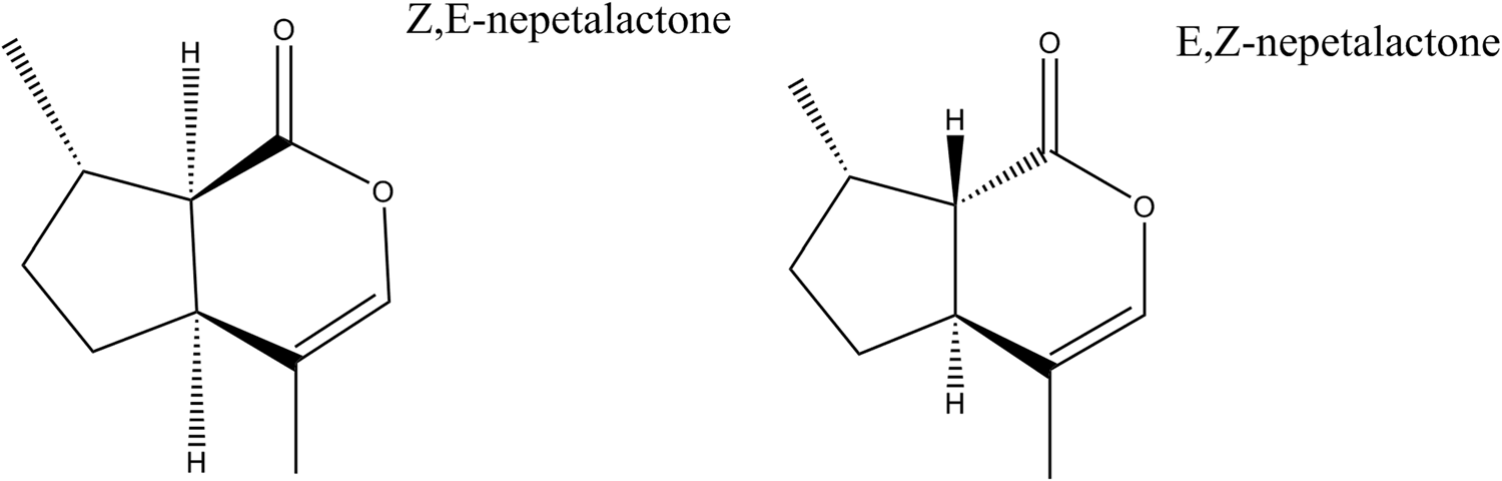
Chemical structures of the two main nepetalactones in *Nepeta cataria*. The structures of Z, E-nepetalactone and E, Z-nepetalactone, the two nepetalactone isomers investigated for repellency activity.

Insects can often discriminate different isomers, perceiving them differently from one another and the stereochemistry of an insect repellent compound can alter its efficacy as a repellent^50,51^. Members of the *Nepeta* genus produce many different stereoisomers of nepetalactone within their essential oils (Fig. 1)^52–54^. Efforts to investigate the differences in the two main crude oil chemotypes (Z, E-nepetalactone dominated or E, Z-nepetalactone dominated) and the isomers of nepetalactone with respect to repellency in mosquitoes have been investigated^55,56^. A World Health Organization (WHO) approved topical application bioassay showed the different essential oil chemotypes were comparable to DEET at repelling *Anopheles gambiae* in a forearm assay from a steam distilled product^56,57^. The Z, E- and E, Z-nepetalactones showed similar efficacy at repelling *A. gambiae* in another WHO approved topical application bioassay where the crude oils and purified compounds were not statistically different from one another at repelling the mosquitoes^56,57^. Results from a biting deterrent assay with *A. aegypti* showed no differences in the crude oils and purified nepetalactones in the ability to stop feeding, however a dose response curve was not generated and the duration of repellency was not investigated^55^. *Nepeta cataria* essential oil was tested for acute oral, dermal and inhalation toxicity and the results showed that *N. cataria* is safe for human use according to the United States Environmental Protection Agency but may cause mild skin irritation^58^.

Due to the need for effective safe insect repellents and since the essential oils and purified compounds of *N. cataria* have shown to be effective at repelling mosquitoes, we investigated several different established nepetalactone based chemotypes of *N. cataria*, their respective purified isomers and compared them to DEET to generate a landing reduction dose response repellency curve. We also conducted a time course analysis of a crude essential oil extract compared to DEET over a 24 hr period to assess repellency.

## Materials and Methods

### Equipment and Chemical Reagents

Bug Dorm insect rearing cages (29.9 cm^3^) were obtained from BioQuip Products (Rancho Dominguez, CA). A HotHands hand warmer was used as the heat source and these were procured from Kobayashi LLC (Dalton, GA). Crude commercial catnip oil was obtained from Plant Therapy Essential Oils (Twin Falls, ID). TetraMin Tropical Tablets used for mosquito rearing were purchased from Tetra (Blacksburg, VA). FisherBrand filter paper circles (18.5 cm; 09-795 G) were obtained from Fisher Scientific Co. (Fair Lawn, NJ).

Chromatographic grade methyl tert-butyl ether (MTBE), as well as reagent grade acetone and DEET at 97% purity were obtained from Sigma Aldrich (St. Louis, MO). Anhydrous sodium sulfate (Na_2_SO_4_) and chromatographic grade hexane and ethyl acetate (EtOAc) were purchased from Fisher Scientific Co. (Fair Lawn, NJ). Chromatographic grade helium was obtained from Airgas, Inc. (Radnor, PA).

### *Nepeta cataria* Cultivation and Essential Oil Preparation

The clonal populations serving as source material for the essential oils includes two new and unique Rutgers University catnip cultivars *N. cataria* cv. ‘CR3’ and *N. cataria* cv. ‘CR9’ both stable in unique essential oil chemistry, promising growth characteristics and high essential oil yields^40^. The CR3 essential oil chemotype is mainly comprised of the E, Z-nepetalactone isomer yet also produces Z, E-nepetalactone. The CR9 essential oil chemotype is dominated by the Z, E-nepetalactone isomer and produces little E, Z-nepetalactone. The hydro-distilled essential oil from these two populations served as the source for the crude essential oil treatments and partitioned for subsequent fractionation and nepetalactone purification.

These two genetically distinct populations were propagated at the Rutgers University Research Greenhouses (New Brunswick, NJ) where vegetative clones were made by making cuttings at the terminal nodes and briefly dipping them in Hormodin 2, 0.3% indole-3-butyric acid (IBA) to induce root formation and placed in a mist house until roots developed. The clones were then transplanted on June 6^th^, 2015 to the Rutgers University Clifford E. & Melda C. Snyder Research and Extension Farm in Pittstown, NJ. Just before the plants were in full flower, they were harvested and dried at 37 °C with an onsite Powell walk-in forced air heat dryer. Once the plants had lost all moisture, the leaves and flowers were separated from the stems before hydro-distillation. Essential oils were extracted by hydro-distilling 60 g of dried *N. cataria* leaves and flowers in a 2 L round bottom flask for 3 hours in 1 L of water and the essential oil was collected in a Clevenger-type trap.

### GC/MS Sample Preparation and Injection Conditions

Essential oil samples were prepared by the extraction of 10 µL of crude *N. cataria* essential oil with 1.5 ml of MTBE, which was then dried with Na_2_SO_4_ and centrifuged at 13 Krpm. The supernatant was transferred to a sampling vial for analysis. Essential oil separation was performed on a Shimadzu 2010 Plus gas chromatograph equipped with an AOC-6000 auto-sampler. The analysis of the relative abundance of compound fragments was performed on a Shimadzu TQ8040 MS.

An injection volume of 1 µL was separated using chromatographic grade helium on a H-Rxi-5Sil MS column held at 35 °C for 4 min then heated to 250 °C at 20 °C/min then held for 1.25 min at 250 °C. The inlet temperature was 250 °C with a splitless injection. The ion source temperature was set at 200 °C, the interface temperature was set at 250 °C, the solvent cut time was 3.5 min, and the detector voltage was set to 0.2 kV with a threshold of 1000. Peak integration percentages were generated using the GCMSsolution v4.3© software from Shimadzu Corporation. Individual identities were determined by comparing the mass spectral results to current literature and screening them in the NIST05.LIB, NIST05s.LIB, W10N14.lib and the W10N14R.lib mass spectral libraries.

### Purification of Nepetalactones

Approximately 12 grams of the different catnip essential oils were repeatedly chromatographed on a silica gel column using a stepwise gradient of hexane-EtOAc from 100%:0% to 80%:20%. CR9 crude essential oil was used for *Z, E*-nepetalactone purification and CR3 crude essential oil was used for *E, Z*-nepetalactone purification. Achieved fractions containing the target components were monitored by silica H TLC (hexane-EtOAc/90%:10%) and LC–MS obtaining Z, E-nepetalactone (800 mg) and E, Z-nepetalactone (200 mg). For LC-MS analysis, a Hewlett Packard Agilent 1100 Series LC/MS (Agilent Technologies, Waldbronn, Germany) equipped with an autosampler, quaternary pump system, DAD, degasser, MSD trap with an electrospray ion source, and software for data processing (HP ChemStation) was applied. The structures of these two purified compounds were determined and verified by UV, MS and NMR spectrometric methods, the latter using a Bruker NMR 400 MHz, and in comparison with references^59^.

### Mosquito Rearing

*Aedes aegypti* (*wt*. Orlando) eggs were reared in water at 27 °C with 80% humidity under a 12-hour day/night light cycle and were transferred during the rearing process with an eyedropper. General fish food tablets were used as the energy source for the maturing mosquitoes. The *A. aegypti* eggs were placed in a container holding water and once hatched and formed into larvae, they were separated from the unhatched eggs and placed into fresh water. As the mosquitoes began to form into pupae, they were separated from the smaller less developed larvae and placed into fresh water. This container was then placed into a rearing cage where the pupae were allowed to mature into adults. Mature females were visually identified, then separated out of the population by aspirating them into a separate rearing cage where they were given a 10% sucrose solution as an energy source. Mature females were kept at these conditions until experimentation.

### Dose Dependent Curve Generation

Repellency was determined by a one-choice landing assay that uses the amount of mosquito landings to calculate the overall effectiveness of a candidate repellent when compared to a control. Twenty, adult female *A. aegypti* mosquitoes were aspirated into a rearing cage and starved for 1 day. Testing was performed between 10:00 am and 4:00 pm PST during *A. aegypti’s* active hours. A 37 °C heat pack was used to as the heat source to attract the mosquitoes in the upper region of the back panel of the rearing cage. The different treatments were extracted in acetone and 500 µL applied to the filter paper (6 × 9 cm) used to wrap the heat source. A control of acetone was applied to filter paper before and after each treatment to ensure reproducibility in mosquito behavior where percent repellencies for each treatment represented the percent reduction in mosquito landings from the prior control. The controls showed no significant repellency at all and thus data is not presented. Six repetitions we performed with twenty mosquitoes for each treatment and at each concentration along with an acetone control. The dose response curve was generated from identifying a concentration of the treatments that exhibited complete repellency and then working in reverse logarithmically with respect to the concentration of the treatment. Initial tests showed that few enough landings were observed at 1.0% generating a 95% reduction in mosquito landings and was defined as complete repellency. Time-lapse photography recorded one image every five seconds for five minutes where a custom macro named “Final Mosquito Counter” generated a Z stack of the images and counted the number of mosquitoes on the filter paper wrapped heat pack automatically through the open source image processing software ImageJ^60^.

### Time Course Assay

This assay was performed the same way as the dose response curve, except we were evaluating how long the essential oils can be an effective repellent. During a 24 hr period, the efficacy of a 10% CR9 essential oil and DEET treatment was monitored at 0 hr, 1 hr, 2 hr, 4 hr, 8 hr and 24 hr intervals. Six repetitions were done for each treatment where the mosquito landings were counted and analyzed similarly to the dose response curve method and at each time point along with an acetone control before and after to ensure mosquito behavior reproducibility where percent repellencies for each treatment represented the percent reduction in mosquito landings from the prior control. After the acetone treatment was applied to the filter paper and the 0 hr treatment sample was done, the filter paper was collected and used in the subsequent time intervals.

### Statistical Analysis

Values are presented as means ± standard deviation (SD). Data was analyzed by an unpaired, two-tailed student’s t-test to identify significant differences (P < 0.05).

## Results

The MS for both *Z, E*-nepetalactone and *E, Z*-nepetalactone showed [M + H]^+^ 167. NMR results for *Z, E*-nepetalactone showed: ^1 ^H NMR (CDCl_3_) δ 6.19 (1 H, s), 2.76 (1 H, dd, J = 8.0 Hz), 2.45 (1 H, dd, J = 8.0 Hz), 2.40 (1 H, m), 2.04 (1 H, m), 1.92 (1 H, m), 1.64 (3 H, s), 1.55 (1 H, m), 1.28 (1 H, m), 1.22 (3 H, d, J = 8.0 Hz); ^13 ^C NMR (CDCl_3_) δ 170.9 (C1), 133.6 (C2), 115.3 (C3), 49.4 (C7a), 40.8 (C4a), 39.8 (C7), 33.0 (C6), 30.9 (C5), 20.3 (C9), 15.5 (C8). NMR results for *E, Z*-nepetalactone showed: ^1 ^H NMR (CDCl3) *δ* 6.23 (1 H, s), 2.70 (1 H, m), 2.48 (1 H, m), 2.35 (1 H, m), 2.16 (1 H, m), 1.97 (1 H, m), 1.73 (3 H, s), 1.38 (2 H, m), 1.14 (3 H, d, *J* = 7.0 Hz); ^13 ^C NMR (CDCl_3_) *δ* 170.2 (C1), 135.9 (C2), 120.4 (C3), 49.1 (C7a), 37.3 (C4a), 32.1 (C6), 30.0 (C7), 26.1 (C5), 17.6 (C9), 14.3 (C8).

**Figure 2:**
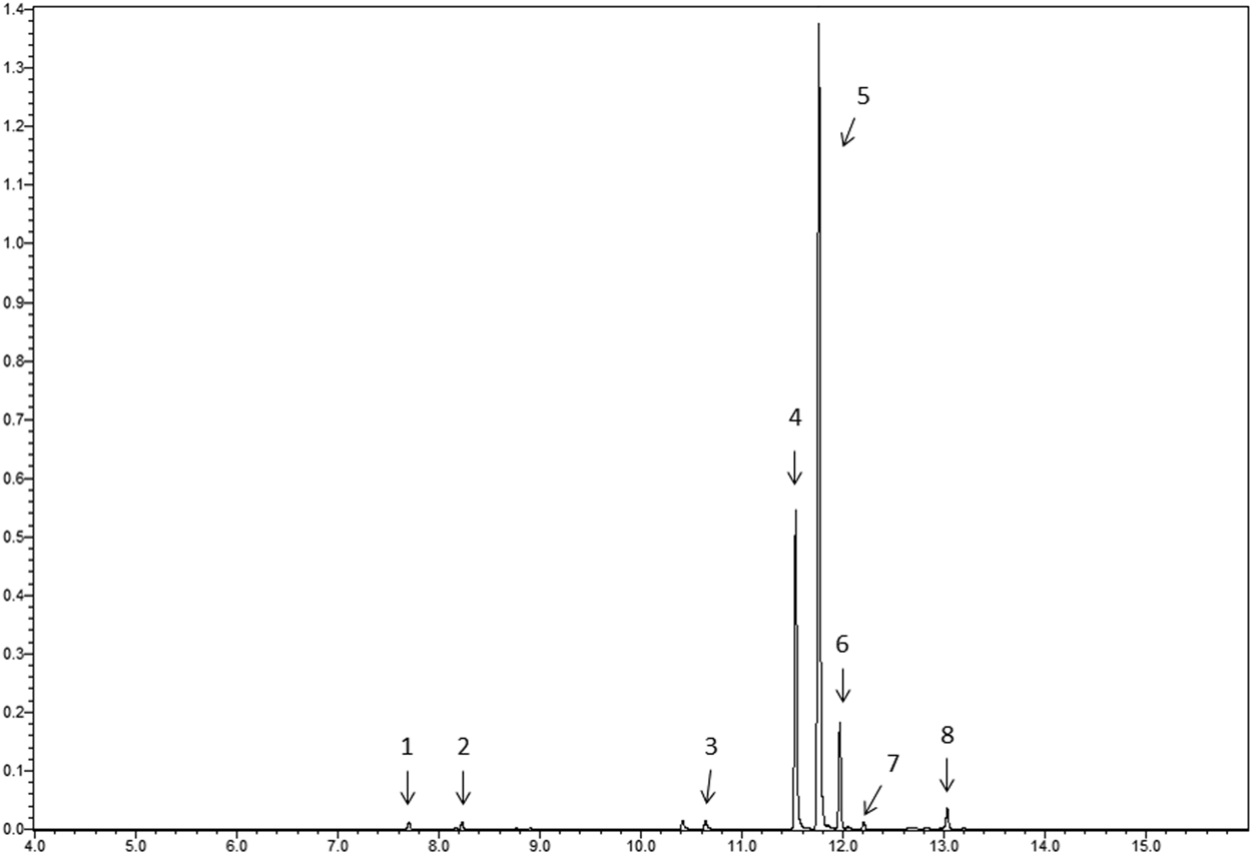
Representative *Nepeta cataria* essential oil chromatogram. Commercial *N. cataria* essential oil chromatogram showing the separated compounds contained in all the crude essential oil treatments. Compound identification numbers correspond to Table 1.

In terms of nepetalactone production, the CR3 chemotype contained 68.2% E, Z-nepetalactone and 25.6% Z, E-nepetalactone while CR9 contained 90.1% Z, E-nepetalactone and less than 0.5% E, Z-nepetalactone and the commercial *N. cataria* essential oil contained 24.4% Z, E-nepetalactone and 62.8% E, Z-nepetalactone (Table 1; Fig. 2). Other minor volatile essential oil components included in catnip samples CR3, CR9 and the commercial oil are β-pinene at <0.5%, 0.58% and 0.57% respectively, β-caryophyllene at 1.57%, 4.72% and 8.11% respectively, humulene at <0.5%, 0.51%, and 0.58% respectively and caryophyllene oxide at 1.27%, 5.79%, and 1.56% respectively. Compounds detected in just the commercial sample were α-pinene and carvone at concentrations of 0.52% and 0.67% respectively (all as rel. % of total essential oil).

**Table 1:** Chemical profile of *Nepeta cataria* crude essential oil treatments.

ID #	Essential oil Constituent	Mass	R_T_ (min)	CR3 Oil Peak Area %	CR9 Oil Peak Area %	CO Oil Peak Area %
1	α-Pinene	136	7.702	ND	ND	0.52
2	β-Pinene	136	8.227	T	0.58	0.57
3	Carvone	150	10.638	ND	ND	0.67
4	Z, E Nepetalactone	166	11.536	27.45	85.33	24.34
5	E, Z Nepetalactone	166	11.75	69.37	1.68	62.62
6	β-carophyllene	204	11.964	1.57	4.72	8.11
7	Humulene	189	12.201	T	0.51	0.58
8	Caryophyllene Oxide	187	13.028	1.27	5.79	1.56

The eight essential oil constituents that represent >98% of the overall peak area detected in the three essential oil treatments (catnip line CR3, cultivar CR9 and a commercial *N. cataria* essential oil) including their mass, retention time and peak area percentages.

T: Trace level of compound where overall concentration was less than 0.5%.

ND: Compounds not detected in the sample.

**Figure 3:**
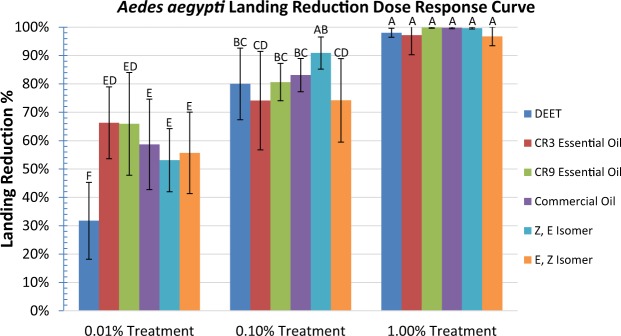
Landing reduction dose-response for *Aedes aegypti* mosquitoes. Dose response landing reduction for *Aedes aegypti* using DEET, *N. cataria* CR3 crude essential oil, *N. cataria* cv. ‘CR9’ crude essential oil, a commercial *N. cataria* essential oil, purified Z, E-nepetalactone and E, Z-nepetalactone treatments at 0.01%, 0.10% and 1.00%. Values are presented as means ± standard deviation (SD). Data was analyzed by an unpaired, two-tailed student’s t-test to identify significant differences (P < 0.05).

**Figure 4:**
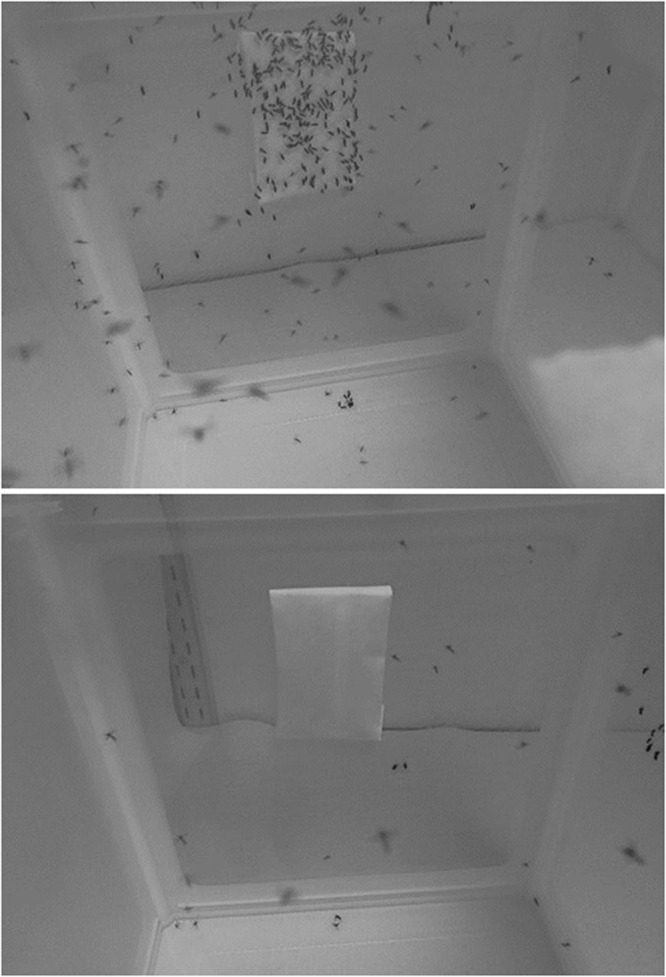
Representative heat packs showing landing areas before and after *Nepeta cataria* treatments. Z stacks consisting of 60 images taken at 5 second intervals over 5 minutes showing the inside of a rearing cage and the heat source during experimentation with 20 female *Aedes aegypti* mosquitoes. A: Representative Z stack of control treatment. B: Representative Z stack of 1.00% *N. cataria* treatments.

Female *Aedes aegypti* mosquitoes are attracted to a human body temperature (37 C degree) source and repellency of different chemicals can be conveniently tested by measuring the reduction in numbers of mosquitoes attracted to the heat source. In the dose response curve, DEET and all of the catnip treatments at 1.00%, decreased the landings of mosquitoes by >95% and grouped together statistically for repellency (Figs. 3 and 4). At 0.01%, 0.10% and 1.00% DEET repelled 31.7% ± 13.5, 80.0% ± 12.6 and 98.0 ± 1.5 of the mosquitoes respectively. At 0.01%, 0.10% and 1.00% the CR3 crude essential oil repelled 66.3% ± 12.7, 74.1% ± 17.3 and 97.2% ± 6.9 of the mosquitoes respectively. At 0.01%, 0.10% and 1.00% the CR9 crude essential oil repelled 65.9% ± 18.1, 80.6% ± 6.6 and 99.8% ± 0.2 respectively. At 0.01%, 0.10% and 1.00% the commercial essential oil repelled 58.7% ± 16.0, 83.1% ± 5.9 and 99.8% ± 0.3 respectively. At 0.01%, 0.10% and 1.00% the purified Z, E isomer repelled 53.1% ± 11.1, 90.9% ± 5.7 and 99.7% ± 0.3 of the *A. aegypti* mosquitoes respectively and the purified E, Z isomer repelled 55.7% ± 14.4, 74.2% ± 14.7 and 96.8% ± 3.3 respectively.

**Figure 5:**
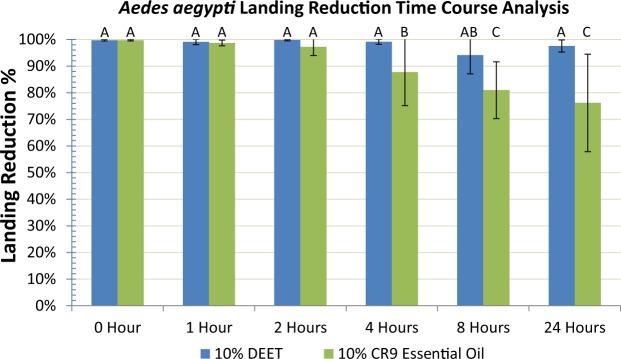
Landing reduction time course analysis for *Aedes aegypti* mosquitoes. Time course analysis comparing the landing reduction percentages of *Aedes aegypti* mosquitoes with 10% DEET and crude CR9 essential oil treatments over 24 hours. The samples were tested at 0 hour, 1 hour, 2 hours, 4 hours, 8 hours and 24 hours after application of the treatments. Values are presented as means ± standard deviation (SD). Data was analyzed by an unpaired, two-tailed student’s t-test to identify significant differences (P < 0.05).

In the time course assay a higher concentration (10%) was used to more closely match the bioactive repellents found in commercially available formulations of insect repellents. The 10% CR9 essential oil treatment generated >95% repellency for the first two hours and was statistically similar to DEET (Fig. 5). At the 0 hr, 1 hr, 2 hr, 4 hr, 8 hr and 24 hour time intervals after treatment, DEET repelled 99.8% ± 0.3, 99.1% ± 1.0, 99.8% ± 0.3, 99.1% ± 1.0, 94.1% ± 7.0 and 97.5% ± 2.3 mosquitoes respectively. At the 0 hr, 1 hr, 2 hr, 4 hr, 8 hr and 24 hour time intervals after treatment, CR9 essential oil repelled 99.7% ± 0.2, 98.7% ± 1.1, 97.2% ± 3.2, 87.8% ± 12.6, 81.0% ± 10.7, and 76.2% ± 18.3 mosquitoes respectively.

## Discussion

The data presented herein demonstrates that the essential oils of *N. cataria* can serve as a natural source for an effective *A. aegypti* repellent that is comparable to DEET for the first two hours after application but needs to be reapplied to maintain complete repellency from *A. aegypti* mosquitoes. Since the two main chemotypes of the essential oils (CR3, CR9) were not statistically different from one another and DEET at 1.00%, individuals who are in need of an immediately effective insect repellent could use either *N. cataria* essential oil chemotype for protection and for two hours of protection they can use the ‘CR9’ chemotype of *N. cataria*. The data from the 0.01% treatment in the dose response curve is in contrast to a biting deterrent study in which DEET was shown to be more effective than either of the nepetalactones and can be attributed to the differences in the tests and/or mosquitoes. However, results from the time course assay demonstrate that the CR9 essential oil is as effective as DEET for the first two hours after application in the landing reduction assay. The two isomers were equally effective at repelling mosquitoes so repellents made from these will be effective as long as it has a high enough concentration of either nepetalactone isomer. While the 10.00% solution of the CR9 chemotype is just as effective as DEET for the first 2hrs, a reapplication would be required after that to keep the numbers of landings reduced by 95% or greater. Efforts to increase the effective repellency duration of the catnip essential oils through formulation development should be considered so that the repellent maintains the >95% landing reductions for over 2hrs so reapplication would be less frequent.

*Nepeta* species producing nepetalactones are found throughout in many regions and can be cultivated in additional areas where their essential oil can be distilled to repel mosquitoes that inhabit the same regions^49^. The processing of raw plants to yield essential oils is accomplished using a variety of extraction or distillation technologies including solvent, supercritical CO_2_ or water and steam respectively. In rural communities, the same processing technologies now in commercial use that produce a wide range of essential oils can be used to procure the aromatic volatile oils from *N. cataria* in rural regions. These communities can be protected from disease vectoring insects from the plantings of *Nepeta sp*. that contain high amounts nepetalactones in their already established essential oil crop fields. While many catnip and catmint plants produce essential oils and of these many have nepetalactones as a component in their essential oil, overall functional agricultural yields of the total essential oil and/or the concentration of the nepetalactones is low, leading to a commercialization bottleneck. These results are exciting because these two newly developed genetic varieties produce high yields of total essential oil, and of the oil, each are rich in the bioactive nepetalactones overcoming two of the major constraints in developing a new bioactive ingredient, the cost and availability of the raw material. Individuals in rural communities can overcome the costs of purchasing *N. cataria* essential oil by cultivating catnips that are rich in essential oil and nepetalactones and processing the essential oil themselves for protection. In this case, both CR9 and CR3 strains created offer promise to protecting individuals against *A. aegypti* mosquitoes.

## Data Availability

The datasets generated during the current study are available from the corresponding author upon reasonable request.
